# Association between serum sodium level within normal range and handgrip strength in relation to hypertension status: a cross-sectional study

**DOI:** 10.1038/s41598-020-80163-x

**Published:** 2021-01-13

**Authors:** Yuji Shimizu, Hirotomo Yamanashi, Shoichi Fukui, Shin-Ya Kawashiri, Yasuhiro Nagata, Takahiro Maeda

**Affiliations:** 1 Department of Community Medicine, Nagasaki University Graduate School of Biomedical Sciences, Nagasaki-shi, Sakamoto 1-12-4, Nagasaki, 852-8523 Japan; 2 Department of Cardiovascular Disease Prevention, Osaka Center for Cancer and Cardiovascular Diseases Prevention, Osaka, Japan; 3 Department of General Medicine, Nagasaki University Hospital, Nagasaki, Japan; 4 Center for Comprehensive Community Care Education, Nagasaki University Graduate School of Biomedical Sciences, Nagasaki, Japan; 5 Department of Island and Community Medicine, Nagasaki University Graduate School of Biomedical Sciences, Nagasaki, Japan

**Keywords:** Biomarkers, Health care, Risk factors

## Abstract

Serum sodium concentration within the normal range could act as an indicator of age-related changes such as decrease in muscle strength and impairment of capillary function. Since endothelial injury stimulates endothelial repair by enhancing CD34-positive cell production, the level of serum sodium may be inversely associated with that of circulating CD34-positive cells, thus indicating the degree of age-related endothelial injury. We conducted a cross-sectional study of 246 elderly Japanese men aged 60–69 years. Subjects were stratified by hypertension status because hypertension should act as a strong confounding factor for the analyses performed in this study. Serum sodium concentration was positively associated with handgrip strength in non-hypertensive subjects [standardized parameter estimate (β) = 0.29; p = 0.003], but not for hypertensive subjects (β = 0.01; p = 0.878), while it was inversely associated with circulating CD34-positive cell levels in non-hypertensive subjects [simple correlation coefficient (r) = − 0.28; p = 0.002] but not for hypertensive subjects (r = − 0.07; p = 0.454). For non-hypertensive elderly subjects, serum sodium concentration within the normal range is positively associated with handgrip strength and inversely associated with CD34-positive cells, thus partly indicating the degree of age-related endothelium injury. These associations could prove to be an efficient tool for clarifying the background mechanism governing the decrease in age-related muscle strength.

## Introduction

Elderly men with low serum sodium levels within the normal range (136–138 mEq/L) showed higher risks of major cardiovascular events and total mortality than those with low serum sodium levels within the upper normal range (139–143 mEq/L)^1^. This study also showed a positive association between serum sodium level and muscle mass evaluated on the basis of mid-arm muscle circumference. Since hyponatremia is a multifactorial condition related to aging^2–7^, a serum sodium level within the normal range could act as an indicator of age-related change.

Capillary function, known as a function of microcirculation is associated with cardiovascular risk factors^8,9^.

In addition, sarcopenia is associated with impairment of capillary function^10^ and handgrip strength, a predictor of age-related disability, is an efficient tool for evaluating the decrease in muscle strength and function in the elderly^11^.

Serum sodium levels within normal range could therefore also be positively associated with handgrip strength by indicating age-related disruption of microcirculation.

Further, CD34-positive cell concentration is a known factor contributing to endothelial repair^12^, while endothelial injury in turn should stimulate CD34-positive cell production^13–16^. Serum sodium levels can therefore be inversely associated with CD34-positive cell concentration because higher serum sodium levels might indicate a reduction in endothelial injury, resulting in diminished production of CD34-positive cells.

However, hypertension is well known to be a strong endothelium impairment factor^17^ that induces the reduction of circulating CD34-positive cells due to consumption^18–20^. Consequently, hypertension could act as a strong confounding factor on those associations. To clarify those associations, we conducted a cross-sectional study of 246 Japanese elderly men aged 60–69 who participated in an annual health check-up in 2014–2015.

## Methods

### Study population

The total number of male residents of Goto city aged 60–69 (estimated by the National Institute of Population and Social Security Research in March 2013) was 3264 in 2015^21^. The study population comprised 274 male residents aged 60–69 years from the Goto city located in the western part of Japan, who underwent an annual medical check-up between 2014 and 2015 as recommended by the Japanese government. Subjects with high (> 145 mEq/L, n = 10) and low (< 135 mEq/L, n = 1) sodium levels were excluded from the study population. To avoid the influence of chronic inflammatory disease and paralysis caused by stroke, subjects with a high white blood cell count (WBC) (≥ 10,000 cells/μL) (n = 1) and history of stroke (n = 13) were also excluded from the analysis. Subjects without handgrip strength data (n = 3) were also excluded. The remaining patients, 246 men with a mean age of 65.4 years (standard deviation): 2.6; range 60–69) were enrolled in the study.

All procedures performed in studies involving human participants were in accordance with the ethical standards of the institutional research committee and with the 1964 Helsinki declaration and its later amendments or comparable ethical standards. This study was approved by the Ethics Committee for Human Use of Nagasaki University (project registration number 14051404). Written consent forms were available in Japanese to ensure comprehensive understanding of the study objectives, and informed consent was provided by the participants.

### Data collection and laboratory measurements

Body weight and height were measured with an automatic body composition analyzer (BF-220; Tanita, Tokyo, Japan), followed by calculation of body-mass index (BMI; kg/m^2^). Systolic (SBP) and diastolic blood pressure (DBP) were recorded at rest. Hypertension was defined as a SBP ≥ 140 mmHg and/or DBP ≥ 90 mmHg, as in our previous studies that used circulating CD34-positive cells^18–20^.

Blood samples were collected in an EDTA-2K tube, a heparin sodium tube, a siliconized tube and a sodium fluoride tube. Platelet levels and white blood cells (WBC) in samples from the EDTA-2K tube were measured at SRL, Inc. (Tokyo, Japan) with an automated procedure.

Fresh samples from the heparin sodium tube were used within 24 h of collection to determine the number of circulating CD34-positive cells by means of BD Trucount technology (Beckton Dickinson Biosciences; San Jose, CA), an accurate and reproducible single platform assay conforming to the International Society of Hematotherapy and Graft Engineering (ISHAGE) guidelines^22^ and supported by automated software on the BD FACSCanto II system.

Triglycerides (TG), HDL-cholesterol (HDLc), serum creatinine, γ-glutamyltranspeptidase (γ-GTP), hemoglobin A1c (HbA1c) as well as electrolyte—potassium (K), phosphorus (P), calcium (Ca) and sodium (Na)—concentrations were measured with standard laboratory procedures at SRL, Inc. (Tokyo, Japan). Glomerular filtration rate (GFR) was estimated by means of an established method modified as recently proposed by the working group of the Japanese Chronic Kidney Disease Initiative^23^. With this adapted version, GFR (mL/min/1.73 m^2^) was defines as 194 × (serum creatinine (enzyme method))^−1.094^ × (age)^−0.287^.

Handgrip strength was determined with a handgrip dynamometer (Smedley, Matsumiya Ika Seiki Seisakujo, Tokyo, Japan) as the grip strength from two measurements obtained for each hand, from which the maximum value was used.

### Statistical analysis

Characteristics of the study population stratified by hypertension status were expressed as mean ± standard deviation except for TG, γ-GTP and circulating CD34-positive cells. Since these factors showed a skewed distribution, the characteristics of the study population were expressed as medians [first quartile, third quartile], followed by logarithmic transformation. A χ^2^ test was performed to calculate the p value of each variable on the basis of hypertension status. Simple correlation analysis and multiple linear regression analysis of handgrip strength with sodium concentration or CD34-positive cell levels were performed after adjustments for confounding factors and stratification by hypertension status. For this study it was assumed serum sodium indicates age-related endothelial injury, which stimulates CD34-positive cell production. Therefore, to avoid the influence of over-adjustment on the multiple regression analysis, we performed a separate analysis to evaluate the association of serum sodium concentration and circulating CD34-positive cell levels with handgrip strength. For the multiple linear regression analysis, adjustments were then made for age, SBP (mmHg), antihypertensive medication use (yes/no), BMI (kg/m^2^), HDLc (mg/dL), TG (mg/dL), HbA1c (%) γ-GTP (U/L), WBC (cells/μL), GFR (mL/min/1.73m^2^), K (mEq/L), Ca (mg/dL) and P (mg/dL).

CD34-positive cells constitute a factor that is known to contribute to endothelial repair^12^ in conjunction with platelets^13,14,24,25^, while the number of platelets indicates the activity of vascular repair^18,19^. Furthermore, aggressive endothelial injury could cause reduction in CD34-positive cells due to consumption, but this type of reduction may not affect platelets^18,19,26^. Therefore, we calculated simple correlation coefficients for these factors.

All statistical analyses were performed with the SAS system for Windows (version 9.4: SAS Inc., Cary, NC, USA). Values of p < 0.05 were considered statistically significant.

## Results

Among present study population, 125 are diagnosed as having hypertension.

### Characteristics of study population by hypertension status.

Characteristics of the study population by hypertension status as displayed in Table 1 show that 125 subjects were diagnosed with hypertension. Compared with non-hypertensive subjects, hypertensive subjects showed significantly higher values for systolic blood pressure, diastolic blood pressure, antihypertensive medication use, BMI, and significantly lower values for serum K.

**Table 1:** Characteristics of study population according to hypertension status.

	Hypertension		*p*
	(−)	(+)	
No. of participants	121	125	
Age (years)	65.4 ± 2.5	65.3 ± 2.6	0.770
Systolic blood pressure (SBP) (mmHg)	122 ± 10	149 ± 12	< 0.001
Diastolic blood pressure (DBP) (mmHg)	77 ± 7	93 ± 9	< 0.001
Antihypertensive medication (%)	37.2	55.2	0.005
Body mass index (BMI) (kg/m^2^)	23.3 ± 2.8	24.1 ± 3.1	0.044
Serum HDL-cholesterol (HDLc) (mg/dL)	56 ± 12	58 ± 15	0.230
Serum triglycerides (TG) (mg/dL)	93 [67,124]*^1^	93 [72, 133]*^1^	0.432*^2^
HbA1c (%)	5.7 ± 0.5	5.7 ± 0.7	0.781
Serum γ-glutamyltranspeptidase (γ-GTP) (U/L)	31 [21, 45]*^1^	35 [23, 57]*^1^	0.165*^2^
White blood cells (WBC) (cells/μL)	5592 ± 1409	5844 ± 1496	0.175
Glomerular filtration rate (GFR) (mL/min/1.73m^2^)	73.6 ± 13.7	72.5 ± 13.2	0.539
Serum K (mEq/L)	4.4 ± 0.4	4.3 ± 0.3	0.033
Serum Ca (mg/dL)	9.2 ± 0.3	9.3 ± 0.3	0.327
Serum P (mg/dL)	3.3 ± 0.4	3.2 ± 0.4	0.242
Serum Na (mEq/L)	142.7 ± 1.4	142.6 ± 1.5	0.910
Circulating CD34 positive cells (cells/μL)	0.93 [0.59, 1.47]*^1^	0.99 [0.70, 1.56]*^1^	0.590*^2^
Handgrip strength (kg)	38.1 ± 6.1	39.5 ± 5.8	0.061

Values: mean ± standard deviation. *1: Values are median [first quartile, third quartile]. Regression model for mean values was used for determining p values. *2: Logarithmic transformation was used for evaluating p.

### Correlations between handgrip strength and relevant factors for subjects without hypertension

Results of simple correlation analysis show that handgrip strength is significantly positively associated with serum sodium concentration but not with circulating CD34-positive cell levels for subjects without hypertension. Even with further adjustments for other possible variables, these correlations remain unchanged (Table 2).

**Table 2:** Results of simple correlation analysis and multiple linear regression analysis for subjects without hypertension of handgrips strength with relevant factors adjusted for confounding factors.

	r (p)	CD34-positive cells (+)			CD34-positive cells (−)		
		Serum sodium (−)			Serum sodium (+)		
		Β	β	p	Β	β	p
No. of participants		121			121		
Age	− 0.16 (0.073)	− 0.46	− 0.19	0.060	− 0.47	− 0.20	0.042
SBP	0.08 (0.358)	0.11	0.17	0.070	0.09	0.13	0.149
Antihypertensive medication	− 0.11 (0.226)	− 0.89	− 0.07	0.477	− 0.40	− 0.03	0.743
BMI	− 0.04 (0.682)	0.05	0.02	0.830	0.01	0.005	0.962
HDLc	0.06 (0.520)	0.05	0.10	0.387	0.05	0.10	0.397
TG	− 0.03 (0.717)	1.66	0.13	0.323	1.57	0.12	0.299
HbA1C	− 0.16 (0.080)	− 1.71	− 0.15	0.137	− 1.06	− 0.09	0.346
γ-GTP	− 0.09 (0.306)	− 1.08	− 0.11	0.288	− 0.72	− 0.08	0.460
WBC	− 0.09 (0.338)	− 0.001	− 0.12	0.255	− 0.001	− 0.15	0.130
GFR	− 0.11 (0.242)	− 0.04	− 0.10	0.351	− 0.04	− 0.09	0.357
K	0.08 (0.390)	0.05	0.003	0.976	0.36	0.02	0.817
Ca	0.13 (0.164)	2.84	0.15	0.161	1.37	0.07	0.493
P	0.13 (0.161)	1.23	0.08	0.389	1.82	0.13	0.192
Na	0.32 (< 0.001)	–	–	–	1.29	0.29	0.003
CD34-positive cells	− 0.11 (0.240)	− 1.04	− 0.13	0.222	–	–	–

*SBP* systolic blood pressure, *BMI* body mass index, *HDLc* HDL-cholesterol, *TG* triglycerides, *γ-GTP* γ-glutamyltranspeptidase, *GFR* glomerular filtration rate, *r (p)* simple correlation coefficient (p value), *Β* parameter estimate, *β* standardized parameter estimate, *p* p value for multivariable linear regression models, *Triglycerides* γ-GTP, and CD34-positive cell levels were are calculated as logarithm values.

### Correlations between handgrip strength and relevant factors for subjects with hypertension

Simple correlation analysis findings show that handgrip strength is significantly positively associated with circulating CD34-positive cell levels but not with serum sodium concentration for subjects with hypertension. Even with further adjustments for other possible variables, these correlations remain unchanged (Table 3).

**Table 3:** Results of simple correlation analysis and multiple linear regression analysis for subjects with hypertension of handgrips strength with relevant factors adjusted for confounding factors.

	r (p)	CD34-positive cells (+)			CD34-positive cells (−)		
		Serum sodium (−)			Serum sodium (+)		
		Β	β	p	Β	β	p
No. of participants		125			125		
Age	− 0.30 (0.001)	− 0.71	− 0.32	0.001	− 0.67	− 0.30	0.002
SBP	0.03 (0.755)	0.02	0.04	0.634	0.03	0.06	0.490
Antihypertensive medication	− 0.09 (0.327)	− 0.70	− 0.06	0.545	− 0.66	− 0.06	0.578
BMI	0.09 (0.324)	0.30	0.16	0.106	0.35	0.19	0.071
HDLc	0.09 (0.345)	0.01	0.03	0.766	0.01	0.04	0.724
TG	− 0.07 (0.471)	− 0.95	− 0.09	0.430	− 1.19	− 0.11	0.333
HbA1C	− 0.03 (0.747)	− 0.94	− 0.11	0.235	− 1.10	− 0.13	0.176
γ-GTP	− 0.06 (0.528)	− 0.17	− 0.02	0.843	− 0.49	− 0.06	0.582
WBC	0.07 (0.413)	0.0002	0.04	0.674	0.0004	0.10	0.358
GFR	− 0.12 (0.181)	− 0.03	− 0.08	0.417	− 0.05	− 0.11	0.231
K	0.07 (0.463)	1.59	0.09	0.353	1.65	0.09	0.349
Ca	0.08 (0.362)	0.09	0.01	0.956	0.34	0.02	0.844
P	0.14 (0.115)	1.80	0.13	0.189	2.00	0.14	0.152
Na	0.03 (0.753)	–	–	–	0.06	0.01	0.878
CD34-positive cells	0.24 (0.007)	1.85	0.20	0.031	–	–	–

*SBP* systolic blood pressure, *BMI* body mass index, *HDLc* HDL-cholesterol, *TG* triglycerides, *γ-GTP* γ-glutamyltranspeptidase, *GFR* glomerular filtration rate, *r (p)* simple correlation coefficient (p value), *Β* parameter estimate, *β* standardized parameter estimate, *p* p value for multivariable linear regression models, *Triglycerides* γ-GTP and CD34-positive cell were calculated as logarithm values.

### Simple correlation analysis of circulating CD34-positive cell levels and platelet concentration, and of circulating CD34-positive cell levels and serum sodium concentration by hypertension status

Simple correlation analysis findings demonstrate that for subjects without hypertension, circulating CD34-positive cell levels are significantly positively correlated with platelet concentration but inversely correlated with serum sodium concentration. For subjects with hypertension, no significant correlations were obtained (Table 4).

**Table 4:** Simple correlation analysis of circulating CD34-positive cell with relevant factors.

	Hypertension			
	(−)		(+)	
	r	(p)	r	(p)
No. of participants	121		125	
Platelets	0.30	0.001	0.02	0.866
Na	− 0.28	0.002	− 0.07	0.454

*r* simple correlation coefficient, *(p)* p value. CD34-positive cell levels were calculated as logarithm values.

## Discussion

The major findings of the present study are that for non-hypertensive subjects, independent of known cardiovascular risk factors, serum sodium concentration is significantly positively associated with handgrip strength while significantly inversely associated with circulating CD34-positive cell levels whereas no such significant correlations exist for hypertensive subjects.

A previous Japanese meta-analysis study reported a scatter plot of age compared with handgrip strength based on a meta-regression analysis where handgrip strength (kg) = 78.74 − 0.62 × age (years) for men^27^. As the age range for the present target population was 60–69 years, the reference values of handgrip strength calculated using the above formula ranged from 36.0 to 41.5 kg. The mean (standard deviation) handgrip strength values in all the subjects in the present study were 38.8 (6.0) kg. Therefore, we believe that the representative subjects were secured in the present study population.

A previous study with 3099 elderly men aged 60–79 years without a history of cardiovascular disease (CVD) showed a positive association between serum sodium level and muscle mass^1^. In our study, we found further evidence that independent of known cardiovascular risk factors, the normal range of serum sodium levels is positively associated with handgrip strength limited to elderly men without hypertension.

**Figure 1:**
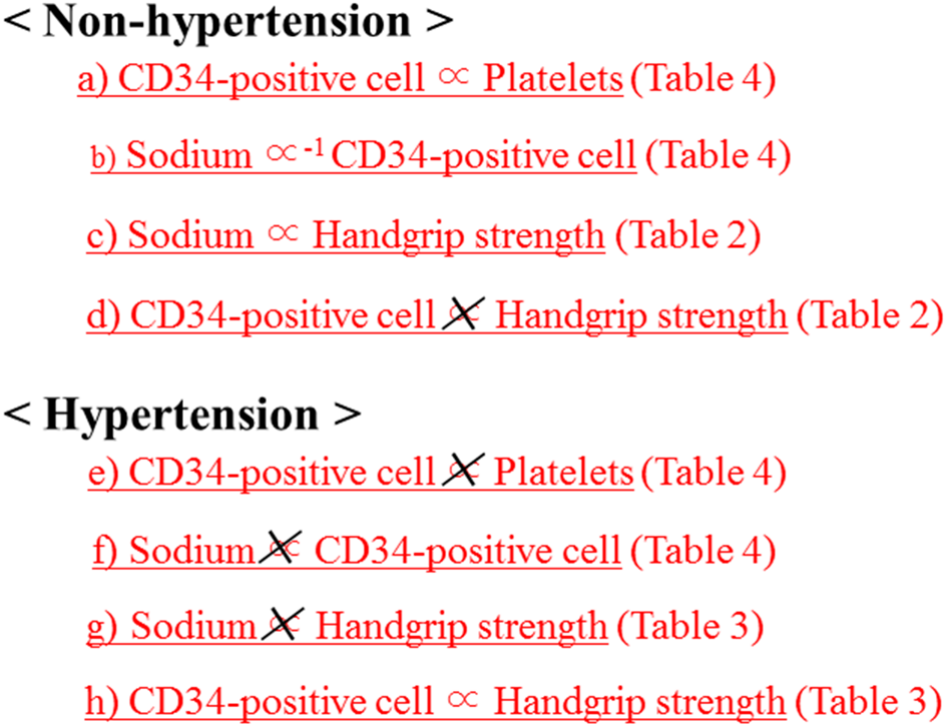
Associations observed in the present study.

**Figure 2:**
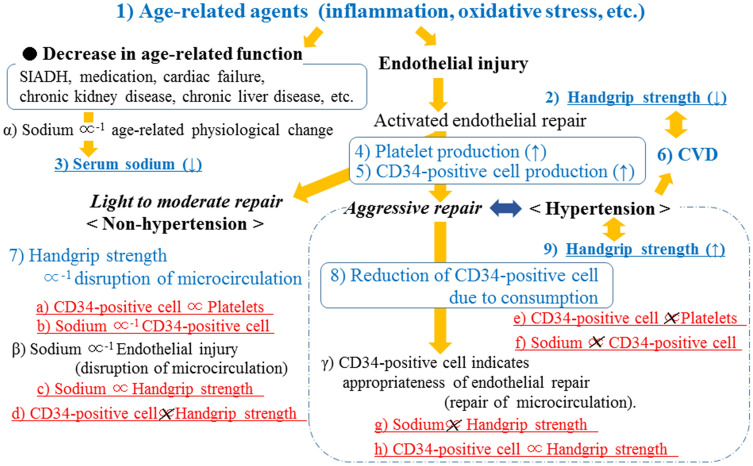
Possible mechanism underlying the association between serum sodium level and handgrip strength.

Figure 1 shows the major findings of the present study that serum sodium level was significantly positively associated with handgrip strength in non-hypertensive subjects but not in hypertensive subjects. In the non-hypertensive subjects, serum sodium level was significantly inversely associated with CD34-positive cells but not in the hypertensive subjects. Figure 2 shows the potential mechanism underlying the present results.

Hyponatremia is the most common electrolyte disorder in the geriatric age group^28^. Even though the most important cause of hyponatremia in the elderly is syndrome of inappropriate secretion of anti-diuretic hormone (SIADH)^2^, hyponatremia is frequently multifactorial^2–7^ and entails several causative factors such as medication (thiazide and loop diuretics, proton pump inhibitors, non-steroidal anti-inflammatory agents), cardiac failure, chronic kidney disease, chronic liver disease, respiratory infections, volume overloaded and dehydration^3,7^ (Fig. 2-3). Since those factors are common in elderly subjects, serum sodium levels within the normal range could function as an indicator of general age-related physiological change instead of indicating a specific disease (Fig. 2-α).

Further, handgrip strength is an efficient tool to evaluate the decrease in muscle strength and function due to its use as a predictor of old disability^11^. Therefore, serum sodium concentration within normal range could be associated with handgrip strength by indicating age-related physical change.

In our study, however, a positive correlation between serum sodium concentration and handgrip strength was observed only for subjects without hypertension (Tables 2, 3, Fig. 2c,g).

Blood flow to the skeletal muscle is a potentially important factor in maintaining muscle function in the elderly^29^, possibly by maintaining protein metabolism^30,31^. Previously, handgrip strength was found to be inversely associated with microcirculation disruption evaluated using the cardiovascular index (CAVI) in subjects with non-hypertension but not in subjects with hypertension^8,32^ (Fig. 2-7).

Hypertension and decreased muscle strength and function are known to be age-related diseases and are reportedly exacerbated by the disruption of the microvascular endothelium and impairment of blood flow resulting from an increase in age-related agents such as inflammatory agents^33^ and oxidative stress^29^ (Fig. 2-1). The condition of the microvascular endothelium might therefore play an important role in the correlations between serum sodium concentration and handgrip strength.

Platelets are the first circulating blood cells that interact with an injured endothelium^34^ (Fig. 2-4). In addition, we reported elsewhere that the number of platelets indicates the activity of vascular repair^18^, while platelet-rich plasma could enhance the proliferation of bone marrow mesenchymal stem cells, which are known to be multi-potent stem cells^25^. Furthermore, CD34-positive cells are known to contribute to endothelial repair^12^ in conjunction with platelets^13,24^ (Fig. 2-5), while the number of circulating CD34-positive cells could indicate the capability of endothelial maintenance^19,20,35^. This means that platelets could be positively associated with circulating CD34-positive cell levels, as observed in our subjects without hypertension (Table 4; Fig. 2a). However, since the production of circulating CD34-positive cells must be stimulated by endothelial injury, the level of circulating CD34-positive cells could indicate the degree of age-related endothelial injury (Fig. 2-5). In our current study, we identified a significant inverse correlation between serum sodium concentration and circulating CD34-positive cell levels in subjects without hypertension (Table 4; Fig. 2b,f). For subjects without hypertension, serum levels of sodium could therefore be inversely associated with age-related endothelial injury (Fig. 2-β).

Furthermore, hypertension is a well-known strong endothelial impairment factor^17^ that causes aggressive endothelium repair, which in turn may cause a reduction in circulating CD34-positive cells due to consumption^18,26^ (Fig. 2-8), although this type of reduction may not affect platelets^18,19,36^. This explains why, even if CD34-positive cell levels are significantly positively associated with platelets in non-hypertensive subjects, no significant correlation was observed for hypertensive subjects (Table 4; Fig. 2a,e).

Reduction of circulating CD34-positive cells due to consumption might therefore determine the number of circulating CD34-positive cells in subjects with hypertension but not in those without hypertension. Lower levels of circulating CD34-positive cells in hypertensive subjects might indicate the existence of aggressive endothelial repair induced by severe endothelial injury, both of which are harmful factors for maintaining muscle strength. As a result, we detected a positive association between handgrip strength and circulating CD34-positive cells in hypertensive but not in non-hypertensive men (Tables 2, 3; Fig. 2d,h), as we did in a previous study^37^. In this case, CD34-positive cell levels indicate the appropriateness of endothelial repair (Fig. 2-γ).

Aggressive endothelial repair, which induces reduction of circulating CD34-positive cells due to consumption, could be associated with hypertension^19^ (Fig. 2-8). However, previous studies revealed a positive association between handgrip strength and blood pressure in older participants^38,39^ (Fig. 2-9). In our study, even if the power did not reach significance, the subjects with hypertension showed slightly stronger handgrip strength than those without hypertension (Table 1). Hypertension is a well-known cardiovascular risk factor^40^ (Fig. 2-6), and low handgrip strength has been reported to be positively associated with CVD^41^ (Fig. 2-2). As CD34-positive cells are positively associated with handgrip strength in hypertension (Table 3; Fig. 2h), active appropriate endothelial repair stimulated by endothelial injury might play an important role in maintaining muscle strength in hypertensive subjects, while low-grade endothelial injury might be critical for maintaining muscle strength in the absence of hypertension. Therefore, even serum sodium levels within the normal range could act as an indicator of general age-related physiological changes in non-hypertensive subjects (Fig. 2-α), including handgrip strength (Table 2; Fig. 2c). Under the influence of hypertension, serum sodium level was no longer associated with handgrip strength because hypertension itself could act as a confounding factor in this association (Table 3; Fig. 2g). A previous study revealed that active endothelial repair should have a beneficial influence on maintaining muscle strength in elderly patients with hypertension^42,43^ that could support this mechanism. Further investigation to clarify the mechanism by which hypertension possesses a beneficial influence on maintaining muscle strength is necessary.

Unlike a general epidemiological study, which reveals the risk factors for the same unfavorable conditions as those of our subjects, our current study was used a multi-faceted analysis of a simple target population. Therefore, our study not only identified the simple risk factors but also clarified the possible background mechanism of age-related physical change.

Although our present study employs a small sample size, it is the largest study in the world that deals with circulating CD34-positive cells among the general population who are selected in a strict manner as like previous of our study^18–20,26,36,37^.

The clinical implication of our findings is that serum sodium concentration, which is a factor measurable in daily clinical practice, could act as an indicator of age-related endothelial injury and a predictor of age-related disability in non-hypertensive subjects.

A few potential limitations of this study warrant consideration. Because the study population comprised subjects who underwent an annual health-checkup, selection bias may exist since the subjects were likely have a higher than average self-interest in their health. Although serum sodium concentration is inversely associated with circulating CD34-positive cell levels among subjects without hypertension, no data were available for the evaluation of endothelial function. As aging is the main contributing factor to decline in endothelial function^44^, further analyses that include endothelial function-related data such as flow-mediated dilation (FMD) will be necessary. Unknown confounding factors such as medication status and duration of medication for hypertension, diabetes, dyslipidemia, inflammatory disease, and thyroid disease, could influence the present study. Further investigation with consideration of this information is necessary.

In conclusions, independent of known cardiovascular risk factors, serum sodium concentration is significantly positively associated with handgrip strength and significantly inversely associated with circulating CD34-positive cell levels for non-hypertensive subjects whereas no such significant correlations were observed for hypertensive subjects.
